# Persistent high antibody titres against *Coxiella burnetii*after acute Q fever not explained by continued exposure to the source of infection: a case-control study

**DOI:** 10.1186/s12879-014-0629-6

**Published:** 2014-11-25

**Authors:** Rana Jajou, Cornelia Christina Henrica Wielders, Monique Leclercq, Jeroen van Leuken, Shahan Shamelian, Nicole Renders, Wim van der Hoek, Peter Schneeberger

**Affiliations:** Department of Medical Microbiology and Infection Control, Jeroen Bosch Hospital, 's-Hertogenbosch, The Netherlands; Centre for Infectious Disease Control, National Institute for Public Health and the Environment, Bilthoven, The Netherlands; Department of Internal Medicine, Bernhoven Hospital, Uden, The Netherlands; Institute for Risk Assessment Sciences, Faculty of Veterinary Sciences, Utrecht University, Utrecht, The Netherlands

**Keywords:** Coxiella burnetii, Q fever, The Netherlands, Chronic, Distance

## Abstract

**Background:**

From 2007 to 2010, (the southern part of) the Netherlands experienced a large Q fever epidemic, with more than 4,000 reported symptomatic cases. Approximately 1 - 5% of the acute Q fever patients develop chronic Q fever. A high IgG antibody titre against phase I of *Coxiella burnetii* during follow-up is considered a marker of chronic Q fever. However, there is uncertainty about the significance and cause of persistence of high IgG phase I antibody titres in patients that do not have any additional manifestations of chronic Q fever. We studied whether continued or repeated exposure to the source of infection could explain elevated IgG phase I antibody levels.

**Methods:**

A case-control study was performed to analyze predictors for possible chronic Q fever. Possible chronic Q fever cases (n = 53) are patients with phase I IgG antibody titre ≥1:1,024 at any point in the 9 - 18 months after acute Q fever diagnosis, with a negative PCR test result for *C. burnetii* DNA and without other disease manifestations. Controls (n = 110) are acute Q fever patients that did not develop chronic Q fever, and who consistently had phase I IgG antibody titre <1:1,024 during the 9 - 18 months follow-up. Binary logistic regression was performed to analyze the effect of living close to an infected farm on the high antibody titres. A longitudinal analysis described the serological profiles of cases and controls.

**Results:**

Proximity to infected farms and contact with animal placental material were not associated with an increased risk for possible chronic Q fever. Possible chronic Q fever patients have high IgG phase II as well as IgG phase I antibody titres, even after 48 months of follow-up.

**Conclusion:**

We were unable to explain the cause of persistent high IgG phase I titres among possible chronic Q fever patients by being continuously exposed to the source of infection.

**Electronic supplementary material:**

The online version of this article (doi:10.1186/s12879-014-0629-6) contains supplementary material, which is available to authorized users.

## Background

*Coxiella burnetii* is a gram-negative bacterium that causes the zoonotic infectious disease Q fever. The primary reservoirs for *C. burnetii* are mainly goats, sheep and cattle [1]-[3], which secrete the bacterium in their urine, feces, milk or birth products. *C. burnetii* has also been reported in cats, dogs, birds, horses and rodents [1],[2]. Infected humans may develop acute Q fever, which is a mild, self-limiting influenza-like illness that is sometimes complicated with pneumonia or hepatitis. Approximately 1 - 5% of the acute Q fever patients develop chronic Q fever, which is detected months or years after infection [3]-[5]. *C. burnetii* has two antigenic phases: phase I and phase II. High levels of phase II antibodies are found in acute Q fever, whereas in chronic Q fever high levels of IgG phase I antibodies are predominant [6],[7]. Acute Q fever patients with aneurysm, valvular surgery, vascular prosthesis, renal insufficiency, pregnancy, and immunosuppression are at higher risk for developing chronic Q fever [3],[4],[8].

The primary source of infection is the inhalation of contaminated aerosols [1],[9],[10], that originate directly from animals or from a contaminated environment [9],[11]. People working in certain occupations are considered to be highly exposed to *C. burnetii* [1],[8],[12]-[19], which includes livestock farmers, veterinarians, students and personnel of veterinary schools/universities and veterinary hospitals, slaughterhouse workers, and laboratory workers [1],[8],[10],[15]. Being present when animals give birth increases the risk of infection, since high concentrations of bacteria are found in placental material [11],[20]. However, occupational exposure alone cannot explain the Q fever epidemic in the Netherlands. Rather, the highest risk was for people living close to infected dairy goat farms [21]-[23]. *C. burnetii* can survive for months in the environment in a spore-like form, which may be a source of infection for people that participate in outdoor activities [10]. Environmental conditions in the surrounding of an infected farm might play a role in the spread of the infection: dry soil conditions with little vegetation and high particulate matter concentrations in the air are possible risk factors [24]-[26].

During the years 2007 - 2010, (the southern parts of) the Netherlands experienced a major Q fever epidemic, with more than 4,000 reported symptomatic cases. However, the actual incidence of infection is much higher as 50 - 60% of patients have an asymptomatic *C. burnetii* infection [27],[28]. Probably due to veterinary hygienic measures, culling of pregnant goats, and vaccination of goats, the acute Q fever epidemic in the Netherlands stopped. Nevertheless, a rising number of chronic Q fever patients is seen [3],[29]. A Dutch Q fever consensus group has set up criteria for the diagnosis of proven, probable, and possible chronic Q fever (Table 1) [3],[4]. The Dutch chronic Q fever database listed 284 patients with chronic Q fever: 151 patients with proven chronic Q fever, 64 with probable, and 69 with possible chronic Q fever [30]. Distinction between the categories of chronic Q fever is important in order to understand whether treatment needs to be initiated. Frequent monitoring is warranted, which consists of a three monthly clinical and microbiological follow-up. Radiographical imaging (echocardiogram, PET/CT) should be performed when clinical status stagnates or worsens. Possible chronic Q fever patients solely have a phase I IgG antibody titre of ≥1:1,024, without any of the mentioned manifestations in the category probable and proven chronic Q fever. In general, no antibiotic treatment is initiated in these patients when PCR is negative and no risk factors are identified [3],[4].

**Table 1:** **Criteria for diagnosis of chronic Q fever, according to the Dutch Q fever consensus group** [3]

Classification	Definition
*Proven*	• Positive *C. burnetii* in tissue or blood in absence of acute Q fever infection *OR*
	• IFA phase I IgG titer ≥1:1,024 with definite endocarditis according to the revised Duke criteria* *OR*
	• IFA phase I IgG titer ≥1:1,024 with vascular infection diagnosed with PET/CT, CT, MRI or ultrasound testing
*Probable*	IFA phase I IgG titer ≥1:1,024 with one of the following manifestations:
	• Valvular deviation that does not meet the definition of endocarditis according to the Duke criteria
	• Aneurysm, valvular- or vascular prosthesis without an infection on PET/CT, CT, MRI or ultrasound testing
	• Suspicion of osteomyelitis or hepatitis as an expression of chronic Q fever
	• Pregnancy
	• Clinical symptoms of chronic infection (e.g. fever, weight loss, night sweating)
	• Granulomatous infection
	• Immunodeficiency
*Possible*	Solely a phase I IgG ≥1:1,024^¥^, without any of the manifestations mentioned in the categories proven and probable.

*A set of clinical criteria for the diagnosis of infective endocarditis.

^¥^Phase I IgG antibody titer ≥1:1,024 is within the JBZ measured between 9 - 18 months after acute Q fever diagnosis.

There is uncertainty about the cause of persistence of high antibody titres against *C. burnetii* in possible chronic Q fever patients that do not have any additional manifestations. Host factors are likely to be of importance but one of the hypotheses is that continuous exposure to an infection source or boosting causes persistence of high antibody titres. The aims of the current study are (1) to assess whether proximity to the source of infection could predict the persistent high phase I IgG antibody titres among possible chronic Q fever patients; (2) to study the effect of living close to an infected farm on the high levels of phase I and II IgG antibody titres; and (3) to study the serological follow-up profiles of patients without chronic Q fever and possible chronic Q fever patients.

## Methods

### Study design

A case-control study was performed to evaluate whether continued exposure to the source of infection is associated with persistent high phase I IgG antibody titres among possible chronic Q fever patients, and specifically, to study the effect of living close to an infected farm on the high levels of IgG phase I and phase II antibody titres. In addition, a longitudinal cohort study was performed to describe phase I and II IgG antibody titres among patients without chronic Q fever and possible chronic Q fever patients, four years after acute Q fever diagnosis.

### Population data

#### Study population

The study population consisted of Q fever patients, that were recruited from the Jeroen Bosch Hospital (JBH) in 's-Hertogenbosch, Bernhoven Hospital (BH) in Uden, and from the Laboratory for Pathology and Medical Microbiology (PAMM) in Veldhoven. Approval was obtained from the Medical Ethical Committee (METC Brabant) to approach the study population in the context of the serological follow-up study within the JBH (Q-HORT) (reference number: NL35654.028.11). The Q-HORT study included patients ≥18 years, that were diagnosed with acute Q fever in the years 2007 - 2009, with a follow-up sample submitted approximately 12 months after diagnosis. These patients were invited for this follow-up study approximately four years after the acute Q fever diagnosis. Written informed consent was obtained at the time of the Q-HORT study, which also included permission for being contacted for future research. Actual permission of participation in the current study was obtained via oral informed consent before the telephonic interview took place.

#### Cases

Cases (n = 53) are possible chronic Q fever patients, defined as having a serological profile with phase I IgG ≥1:1,024 at any point between the 9 - 18 months after acute Q fever diagnosis; having a PCR-negative test result; and not fulfilling the criteria for probable or proven chronic Q fever (Table 1). Several possible chronic Q fever patients, of whom the date of acute Q fever diagnosis was unknown, were identified as possible chronic Q fever case due to the persistence of high phase I IgG antibody titres. For these patients, the date of acute Q fever was assumed to be one year before the development of the positive serologic profile of phase I IgG ≥1:1,024. Cases were included when diagnosed with possible chronic Q fever in the period from June 2008 until December 2012 and when being ≥18 years of age at the time of acute Q fever diagnosis.

According to the Q fever guideline of the JBH, acute Q fever patients with a high risk for chronic Q fever development were serologically and clinically followed on the 3^th^, 6^th^, 9^th^ and 12^th^ month after acute Q fever diagnosis in order to monitor the development of chronic Q fever. In case of persistence of high levels of phase I and II IgG antibody titers and in the context of the Q-HORT study, several patients from the JBH, BH and PAMM were followed for a longer period of time, which is 24, 36 and 48 months after acute Q fever diagnosis.

#### Controls

Controls (n = 110) are acute Q fever patients who did not develop chronic Q fever and who had phase I IgG <1:1,024 test results between the 9 - 18 months after acute Q fever diagnosis. Controls were randomly selected from participants of the Q-HORT study, until a 1:2 case-control ratio was reached.

#### Data collection

Clinical data was collected from the hospital information systems. Phase I and II IgG data was collected from the laboratory database of the Regional Laboratory of Medical Microbiology and Infection Control of the JBH, that performs Q fever diagnostic tests for the JBH and the BH. The PAMM provided serological data of their possible chronic Q fever patients that were included in this study. Immunofluorescence Assay (IFA; Focus Diagnostics, Cypress, CA, USA) was used to detect IgG antibodies against *C. burnetii* phase I and II antigens.

A telephonic interview was performed to collect information about medical risk factors for chronic Q fever and to identify possible exposure to the source of infection. Source of infection was defined as having occupational exposure (veterinarian, farmer, laboratory worker, slaughterhouse worker, animal transporter) and/or physical and frequent (minimum one time per week) contact with animals (goats, cattle, sheep, cats, dogs, birds, horses, rodents) and/or animal products (fertilizer, hay/straw, placental material, fur/skin/wool) and/or living relatively close to an infected farm from 2007 - 2013.

For the analysis on the effect of proximity of residential addresses to an infected farm on the phase I and II IgG antibody titres, farms were selected that were bulk tank milk positive or that had Q fever-induced abortion problems. The distance of each patient to each infected farm was calculated based on the exact coordinates of the infected farms (provided by the Ministry of Economic Affairs, Agriculture and Innovation) and the coordinates of the six-digit zip codes, i.e. street-level, of the patients (provided by the Municipal Health Services).

### Statistical data analysis

#### Retrospective case-control study

A Chi-square test was used for the univariate analyses for comparison of proportions between cases and controls.

Within the case-control study two separate analyses were performed. The first analysis was a multivariable logistic regression with backward selection method to investigate which variables are predictors of possible chronic Q fever. All variables with a *p*-value <0.20 in the univariate analysis were included in multivariable logistic regression analysis. The second analysis consisted of a binary logistic regression analysis in which the effect of proximity of residential address to an infected farm on the phase I and II IgG titres 9 - 18 months after acute Q fever diagnosis was studied. This second analysis was performed for the case-control study population and for all patients eligible to be invited in the Q-HORT study, consisting of acute Q fever, possible, probable and proven chronic Q fever patients. For both groups the outcome phase I or II IgG antibody titres was dichotomized in <1:1,024 and ≥1:1,024. The living distance to an infected farm was categorized in 0 - 2,000 meter (m), >2,000 - 5,000 m and >5,000 m, according to Schimmer *et al.* [23].

To evaluate the goodness of fit of the prediction model, the -2 Log likelihood and Nagelkerke R Square were calculated. The area under the Receiver Operating Characteristic (ROC) curve and 95% confidence interval (95% CI) were calculated in order to identify how well the prediction model distinguishes acute Q fever patients from possible chronic Q fever patients.

#### Longitudinal cohort study

Serological follow-up data for possible chronic Q fever patients were reported for 24, 36, and 48 months after acute Q fever diagnosis. The Chi-square test was used to identify whether there was a difference at 48 months after acute Q fever diagnosis in phase I and II IgG antibody titres between patients without chronic Q fever and possible chronic Q fever patients. Serological follow-up data were used from existing data from the same study population as the case-control study.

Analyses for both the case-control and longitudinal study were performed using the statistical software IBM SPSS version 19.0.0. *P*-values <0.05 were considered to be statistically significant.

## Results

### Descriptive characteristics

**Figure 1:**
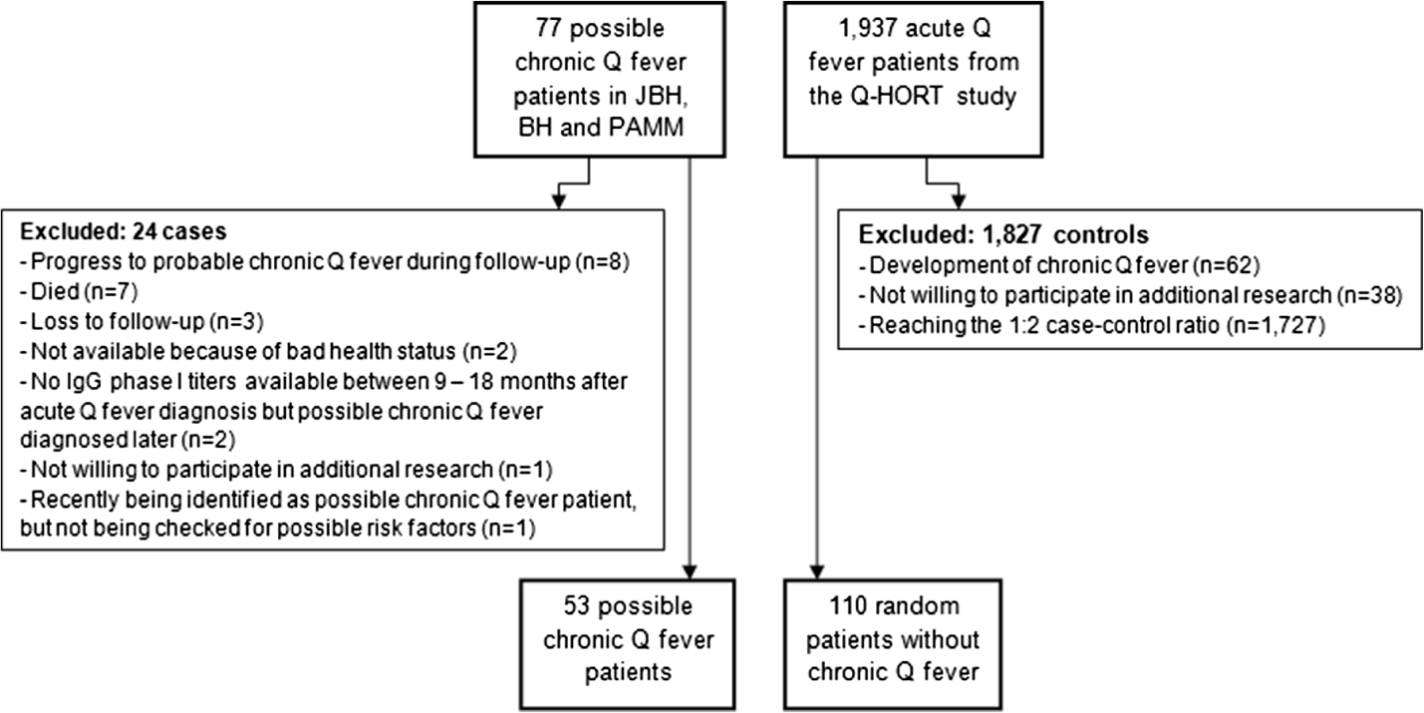
**Inclusion/exclusion criteria for patients without chronic Q fever and possible chronic Q fever patients.** Legend: *JBH: Jeroen Bosch Hospital; BH: Bernhoven Hospital; PAMM: Laboratory for Pathology and Medical Microbiology.

From the 77 eligible possible chronic Q fever patients, 24 patients were excluded because of: progression to probable chronic Q fever during follow-up (n = 8), death (n = 7), loss to follow-up (n = 3), not available for telephonic interview because of bad health status (n = 2), no phase I IgG titres available between 9 - 18 months after acute Q fever diagnosis but possible chronic Q fever diagnosed later (n = 2), not willing to participate in additional research (n = 1) and being recently identified as a possible chronic Q fever patient but not being checked in the clinic for possible risk factors (n = 1) (Figure 1). From the 1,937 acute Q fever patients that were eligible to participate in the Q-HORT study, 62 patients were excluded due to development of chronic Q fever, and 38 due to not willing to participate in additional research (Figure 1).

### Baseline characteristics

In total, 62.3% (n = 33) of the possible chronic Q fever patients and 68.2% (n = 75) of the patients without chronic Q fever were exposed to minimum one of the variables of the source of infection. Except for contact with placental material, which was reported by very few participants, there were no significant differences between cases and controls in baseline characteristics and risk factors (Table 2).

**Table 2:** **Baseline characteristics of study population**

	Possible chronic Q fever ***n = 53***	Non chronic Q fever ***n = 110***	***p-value***
Gender (male)	30 (56.6%)	53 (48.2%)	*0.314*
Mean age ± SD	56.3 (± 10.7)	53.3 (± 12.1)	*0.128*
Living area (rural)	36 (67.9%)	65 (59.1%)	*0.227*
Participant of Q-HORT study	21 (39.6%)	110 (100%)	*-*
Year of acute Q fever diagnosis			*0.454*
2007	4 (7.5%)	24 (21.8%)	
2008	16 (30.2%)	41 (37.3%)	
2009	22 (41.5%)	45 (40.9%)	
2010/2012	2 (3.8%)	0 (0.0%)	
Unknown	9 (17.0%)	0 (0.0%)	
Hospitalization due to Q fever	10 (18.9%)	18 (16.4%)	*0.691*
Smoker	11 (21.2%)	38 (34.9%)	*0.077*
Co-morbidity			
Cardiovascular disease	0 (0%)	7 (6.4%)	*0.060*
Immunosuppressed	3 (5.7%)	5 (4.5%)	*0.758*
Non-hematologic cancer	4 (7.5%)	3 (2.7%)	*0.155*
Pregnancy	1 (1.9%)	4 (3.6%)	*0.544*
Renal failure	1 (2.0%)	0 (0.0%)	*0.141*
Diabetes	5 (9.4%)	6 (5.5%)	*0.343*
Occupational risk	2 (3.8%)	3 (2.7%)	*0.717*
Contact animal products			
Fertilizer	8 (15.1%)	9 (8.2%)	*0.176*
Hay and straw	7 (13.2%)	14 (12.7%)	*0.932*
Placental material	4 (7.5%)	1 (0.9%)	*0.021*
Fur/skin/wool	30 (56.6%)	63 (57.3%)	*0.936*
Intensity contact fertilizer			*0.602*
≤3 times per week	2 (3.8%)	2 (1.8%)	
>3 times per week	5 (9.6%)	6 (5.5%)	
Intensity contact hay and straw			*0.640*
≤3 times per week	2 (3.8%)	7 (6.4%)	
>3 times per week	5 (9.4%)	7 (6.4%)	
Intensity contact placental material			*0.036*
≤3 times per week	1 (1.9%)	1 (0.9%)	
>3 times per week	3 (5.7%)	0 (0.0%)	
Intensity contact fur/skin/wool			*0.446*
≤3 times per week	1 (1.9%)	7 (6.4%)	
>3 times per week	29 (54.7%)	55 (50.0%)	
Contact animals			
Goats, cows or sheep	5 (9.4%)	6 (5.5%)	*0.343*
Cats	13 (24.5%)	19 (17.3%)	*0.275*
Dogs	19 (35.8%)	46 (41.8%)	*0.466*
Birds (including chicken and ducks)	2 (3.8%)	13 (11.8%)	*0.096*
Horses	5 (9.4%)	9 (8.2%)	*0.789*
Rodents	3 (5.7%)	7 (6.4%)	*0.861*
Others (rabbit, fish, pig, tortoise)	2 (3.8%)	10 (9.1%)	*0.659*
Intensity contact animals			*0.877*
≤3 times per week	2 (3.8%)	6 (5.5%)	
>3 times per week	28 (52.8%)	59 (53.6%)	
Distance from house to infected farm			*0.198*
0 - 2,000 m	7 (13.2%)	24 (22.0%)	
>2,000 - 5,000 m	21 (39.6%)	48 (44.0%)	
>5,000 m	25 (47.2%)	37 (33.9%)	

### Multivariable logistic regression analysis

The variables age, smoking, contact with fertilizer, contact with placental material, contact with birds, and living distance to an infected farm had a *p*-value <0.20 in the univariate analysis and were included in multivariable regression analysis. The variable gender had a *p*-value of >0.20 in the univariate analysis, but was included nevertheless due to biological plausibility [18],[21],[31]. The variables cardio-vascular disease, non-hematologic cancer, renal failure, and intensity of the contact with placental material had a *p*-value <0.20 in univariate analysis, but were not included in multivariable regression analysis due to the small numbers (Table 2). The final prediction model did not show any significant association between exposure variables and possible chronic Q fever (Table 3). The best fitting model included the variables smoking, contact with placental material and contact with birds (-2 Log likelihood is 191.7 and Nagelkerke R^2^ = 0.092). The area under the ROC curve (AUC) shows that the final model poorly discriminates between possible chronic Q fever patients and acute Q fever patients that did not develop chronic Q fever (AUC 0.70, 95% CI 0.61 - 0.78, p = 0.001).

**Table 3:** **Univariate and multivariable analysis**

Univariate analysis		
	OR (95% CI)	***p-value***
Age	1.56 (0.81 - 3.01)	*0.186*
Gender	0.71 (0.37 - 1.38)	*0.314*
Smoking	0.50 (0.23 - 1.09)	*0.080*
Contact fertilizer	2.00 (0.72 - 5.51)	*0.182*
Contact placental material	8.90 (0.97 - 81.69)	*0.053*
Contact with birds	0.29 (0.06 - 1.35)	*0.115*
Living distance to infected farm		
<2,000 - 5,000 m	1.50 (0.56 - 4.02)	*0.420*
>5,000 m	2.32 (0.87 - 6.20)	*0.094*
**Multivariable analysis**		
	**OR (95% CI)**	***p-value***
Smoking	0.51 (0.23 - 1.13)	*0.096*
Contact placental material	7.91 (0.85 - 73.99)	*0.070*
Contact with birds	0.30 (0.07 - 1.41)	*0.127*

Within this case-control study, no significant effect was observed for proximity of residential addresses to an infected farm and the phase I and II IgG antibody titres at the 9 - 18 months after acute Q fever diagnosis. Within the entire Q-HORT study population (n = 1,937), phase I IgG antibody titres at the 9 - 18 months after acute Q fever diagnosis were lower for those living >2,000 - 5,000 m from an infected farm compared to the reference group living further away (p = 0.029). This difference was not observed for IgG II antibodies (Table 4).

**Table 4:** **Effect of living distance on the phase I and II IgG antibody titres**

Living distance from infected farm in meters, within case-control study ^†^	phase I IgG <1:1,024 / ≥1:1,024		phase II IgG <1:1,024 / ≥1:1,024	
	OR (95% CI)	***p-value***	OR (95% CI)	***p-value***
>5,000	ref*	ref*	ref*	ref*
>2,000 - 5,000	0.42 (0.16 - 1.11)	*0.079*	0.59 (0.23 - 1.51)	*0.274*
0 - 2,000	0.67 (0.33 - 1.36)	*0.264*	1.00 (0.46- 2.18)	*0.992*
**Living distance from infected farm in meters, within Q-HORT study** ^**¥**^	**phase I IgG <1:1,024 / ≥1:1,024**		**phase II IgG <1:1,024 / ≥1:1,024**	
	**OR (95% CI)**	***p-value***	**OR (95% CI)**	***p-value***
>5,000	ref*	ref*	ref*	ref*
>2,000 - 5,000	0.52 (0.28 - 0.93)	*0.029*	0.98 (0.76 - 1.25)	*0.843*
0 - 2,000	0.65 (0.41 - 1.03)	*0.066*	0.98 (0.79 - 1.22)	*0.857*

*ref = reference category.

^†^Case-control study exists of acute and possible chronic Q fever patients from the Jeroen Bosch Hospital, Bernhoven Hospital and Laboratory for Pathology and Medical Microbiology.

^¥^Q-HORT study exists of acute Q fever, possible, probable and proven chronic Q patients from the Jeroen Bosch Hospital, Bernhoven Hospital and Laboratory for Pathology and Medical Microbiology.

### Serological follow-up

On the 24^th^ and 36^th^ month after acute Q fever diagnosis, the highest frequencies of possible chronic Q fever patients were observed in the category phase I IgG antibody titres 1:1,024 - 1:2,048, and on the 48^th^ month after acute Q fever diagnosis, in the category phase I IgG antibody titres 1:256 - 1:512.

We observed statistically significantly (p = 0.001) more possible chronic Q fever patients than patients without chronic Q fever in the higher categories of phase I and II IgG antibody titres on the 48^th^ month after acute Q fever diagnosis. Among the patients without chronic Q fever, phase I IgG antibody titres remained low (highest frequencies observed in the category 1:32 - 1:128), and phase II IgG antibody titres remained high (highest frequencies observed in the category 1:256 - 1:512) during follow-up (Table 5).

**Table 5:** **Serological follow-up of cases and controls**

Possible chronic Q fever			Possible chronic Q fever		
Antibody titers on the 24 ^th^month after acute Q fever diagnosis	IgG phase I	IgG phase II	Antibody titers on the 36 ^th^month after acute Q fever diagnosis	IgG phase I	IgG phase II
<1:32	0 (0.0%)	0 (0.0%)	<1:32	0 (0.0%)	0 (0.0%)
1:32 - 1:128	2 (7.4%)	0 (0.0%)	1:32 - 1:128	1 (4.8%)	0 (0.0%)
1:256 - 1:512	7 (25.9%)	2 (7.4%)	1:256 - 1:512	7 (33.3%)	3 (14.3%)
1:1,024 - 1:2,048	12 (44.4%)	13 (48.1%)	1:1,024 - 1:2,048	10 (47.6%)	12 (57.1%)
≥1:4,096	6 (22.2%)	12 (44.4%)	≥1:4,096	3 (14.3%)	6 (28.6%)
**IgG phase I**			**IgG phase II**		
**Antibody titers on the 48** ^**th**^ **month after acute Q fever diagnosis**	**Possible chronic Q fever**	**No chronic Q fever** ^**†**^	**Antibody titers on the 48** ^**th**^ **month after acute Q fever diagnosis**	**Possible chronic Q fever**	**No chronic Q fever** ^**†**^
<1:32	1 (2.9%)	55 (51.0%)	<1:32	0 (0.0%)	3 (2.8%)
1:32 - 1:128	6 (17.6%)	47 (43.9%)	1:32 - 1:128	0 (0.0%)	31 (29.0%)
1:256 - 1:512	11 (32.4%)	5 (4.7%)	1:256 - 1:512	5 (14.7%)	51 (47.7%)
1:1,024 - 1:2,048	10 (29.4%)	0 (0.0%)	1:1,024 - 1:2,048	19 (55.9%)	19 (17.8%)
≥1:4,096	6 (17.6%)	0 (0.0%)	≥1:4,096	10 (29.4%)	3 (2.8%)
*p*-value =0.001*			*p*-value =0.001*		

*Chi-square test is used to calculate the *p*-value.

^†^There were three patients without chronic Q fever of which serological data of four years after acute Q fever diagnosis was missing.

## Discussion

The present study provides no evidence that Q fever patients with persistent high phase I IgG antibody levels, classified as possible chronic Q fever, had higher exposure to infection sources than Q fever patients with low phase I IgG titres and thus without chronic Q fever. Continuous boosting of the immune system might not be a major factor in development of possible chronic Q fever, i.e. high phase I IgG antibody titres. This suggests that host-related factors like endocarditis and aneurysm are more important.

Several studies described that occupational exposure or high exposure to the source of infection increases the risk for acquiring Q fever [1],[8],[12]-[19]. However, there are also studies that showed that living close to an infected dairy goat farm increases the risk for acquiring acute Q fever [22],[23],[26] without occupational exposure playing a role [22]. People without direct contact with animals or animal products can also become infected, for example laundry workers that handle contaminated clothing [8],[10]. A study on the risk of acquiring Q fever on a livestock farm concluded that contact with the farm environment rather than contact with a specific farm animal is related to the risk of Q fever [32].

In our case-control study, we did not find an effect of exposure to the source of infection on persistent high phase I IgG antibody titres among possible chronic Q fever patients. We defined exposure to the source of infection as having occupational exposure and/or having at least one time per week physical contact with animals and/or animal products. However, maybe intense contact with one certain animal or animal product might be enough to acquire persistence of high levels of phase I IgG antibody titres.

We also did not find an effect of living relatively close to an infected farm and acquiring possible chronic Q fever. Contradictory, we found a lower risk for those living >2,000 - 5,000 m from an infected farm compared to those living further away. Possibly, distance from residential address to an infected farm is only important for becoming acutely infected and not for the development of high phase I and II IgG antibody titres and therefore the development of possible chronic Q fever.

This study showed that during follow-up, several possible chronic Q fever patients showed persistent high phase I IgG antibody titres up to several years after acute Q fever diagnosis. This should be taken into account when diagnosing a patient with long-term persistence of antibodies as possible chronic Q fever, as described before [33].

The study has several limitations. First, due to the small sample size, only a small number of patients were found to have close and frequent contact with animals and animal products. Therefore, findings (although not significant) of this study should be interpreted with caution. For example, contact with placental material seems to be an important predictor due to the biological plausibility and the high OR of 7.91 found in this study. However, an extremely wide 95% CI was found, reflecting the small number of patients exposed to placental material. It is clear that small sample size is a major limitation of our study. With the available number of cases and controls, and exposure defined as living at 5,000 m or less from an infected farm, the power of the study was only 36%. The study population size was sufficiently powered (80%) to detect an odds ratio of 3 with a significance level of 5%.

This study was performed within an average population that does not have occupational exposure and/or frequent contact with animals and/or animal products. In a population with high occupational exposure (like farmers and veterinarians), having close and frequent contact with animals and animal products is more likely. It cannot be ruled out that high exposure to the source of infection plays a role in persistent high antibody titres in the occupationally exposed, as has also been shown in a study among wool workers in Belgium [34]. An ongoing study among veterinarians with three-year follow-up could possibly clarify this issue. Furthermore, due to the small sample size we were not able to include all possible predictors in the backward selection method. Therefore, we may have missed some relevant predictors for possible chronic Q fever. Based on the research question of this study, the priority of inclusion of variables in multivariable logistic regression analysis was given to variables that included contact with animals and animal products.

Secondly, in our analysis we assumed that a patient might have been become infected by the closest farm to the home address, which not necessarily would have been the case. Also, it is uncertain whether the study population was exposed to the source of infection before the occurrence of their Q fever infection. Therefore, the exact causal relation between patient and farm infection could not be identified, which may explain the non-significant effect we found between living distance to an infected farm and the high levels of phase I and II IgG antibody titres.

Finally, the use of a retrospective telephonic questionnaire as a method of data collection might have caused bias. The questionnaire included questions about exposure to the source of infection from 2007, which may have caused recall bias. However, we expect recall bias to be limited since questions were about being exposed on a regular basis, which is expected to be well reminded by the patient. Also, a telephonic questionnaire may have led to information bias by navigating the answer of the patient to a certain direction in case of doubt. However, compared to a paper questionnaire, a telephonic questionnaire has the benefit of giving a better insight in the degree of exposure over the past years and clarification could be given when a question was misunderstood, which limits information bias. Furthermore, knowing the disease status of the patient at data collection, might have led to information bias. However, by using the same structural questionnaire among the study population, this was less likely to occur.

## Conclusions

Serological follow-up until four years after acute Q fever diagnosis, showed that phase I IgG antibody titres slightly decreased and phase II antibody titres remained high among possible chronic Q fever patients. It is still unclear which factors cause the persistence of high phase I antibody titres among possible chronic Q fever patients. No clear relation could be established between exposure to the source of infection, proximity of residential addresses to an infected farm, and the development of possible chronic Q fever. It is unknown whether possible chronic Q fever patients have actual persistence and replication of *C. burnetii* or whether there still is evidence of boosting. Further research is needed to assess other potential predictors, which could focus on patients genetic tendency to develop high levels of antibodies for a prolonged period of time in response to an infection.

## Authors' contributions

RJ performed the literature review, data collection, statistical analysis and the writing of the manuscript. CCHW participated in the data collection and the design and coordination of the study. ML participated in the data collection. JvL performed distance calculations to infected farms. SS participated in the data collection. NR participated in the data collection and the design of the study. WvdH participated in the design of the study. PS participated in the design and coordination of the study. All authors read and approved the final manuscript.

## Authors’ original submitted files for images

Below are the links to the authors’ original submitted files for images. Authors’ original file for figure 1
