# Supplementary figures and images for: Persistent high antibody titres against Coxiella burnetiiafter acute Q fever not explained by continued exposure to the source of infection: a case-control study
Source: BMC Infect Dis. 2014 Nov 25;14:629. doi: 10.1186/s12879-014-0629-6 (PMC4251683; doi:10.1186/s12879-014-0629-6)

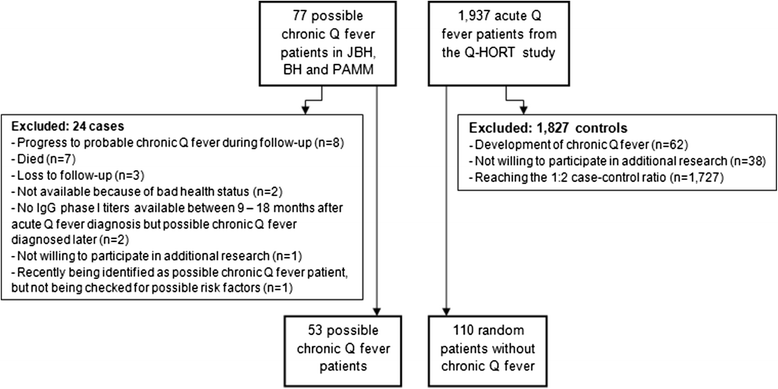

Supplement: Supplementary file 1 — Authors’ original file for figure 1 [file 12879_2014_629_MOESM1_ESM.gif]
